# Patient Perception of Plain-Language Medical Notes Generated Using Artificial Intelligence Software: Pilot Mixed-Methods Study

**DOI:** 10.2196/16670

**Published:** 2020-06-05

**Authors:** Sandeep Bala, Angela Keniston, Marisha Burden

**Affiliations:** 1 College of Medicine University of Central Florida Orlando, FL United States; 2 Division of Hospital Medicine University of Colorado School of Medicine Aurora, CO United States

**Keywords:** artificial intelligence, patient education, natural language processing, OpenNotes, Open Notes, patient-physician relationship, simplified notes, plain-language notes

## Abstract

**Background:**

Clinicians’ time with patients has become increasingly limited due to regulatory burden, documentation and billing, administrative responsibilities, and market forces. These factors limit clinicians’ time to deliver thorough explanations to patients. OpenNotes began as a research initiative exploring the ability of sharing medical notes with patients to help patients understand their health care. Providing patients access to their medical notes has been shown to have many benefits, including improved patient satisfaction and clinical outcomes. OpenNotes has since evolved into a national movement that helps clinicians share notes with patients. However, a significant barrier to the widespread adoption of OpenNotes has been clinicians’ concerns that OpenNotes may cost additional time to correct patient confusion over medical language. Recent advances in artificial intelligence (AI) technology may help resolve this concern by converting medical notes to plain language with minimal time required of clinicians.

**Objective:**

This pilot study assesses patient comprehension and perceived benefits, concerns, and insights regarding an AI-simplified note through comprehension questions and guided interview.

**Methods:**

Synthea, a synthetic patient generator, was used to generate a standardized medical-language patient note which was then simplified using AI software. A multiple-choice comprehension assessment questionnaire was drafted with physician input. Study participants were recruited from inpatients at the University of Colorado Hospital. Participants were randomly assigned to be tested for their comprehension of the standardized medical-language version or AI-generated plain-language version of the patient note. Following this, participants reviewed the opposite version of the note and participated in a guided interview. A Student *t* test was performed to assess for differences in comprehension assessment scores between plain-language and medical-language note groups. Multivariate modeling was performed to assess the impact of demographic variables on comprehension. Interview responses were thematically analyzed.

**Results:**

Twenty patients agreed to participate. The mean number of comprehension assessment questions answered correctly was found to be higher in the plain-language group compared with the medical-language group; however, the Student *t* test was found to be underpowered to determine if this was significant. Age, ethnicity, and health literacy were found to have a significant impact on comprehension scores by multivariate modeling. Thematic analysis of guided interviews highlighted patients’ perceived benefits, concerns, and suggestions regarding such notes. Major themes of benefits were that simplified plain-language notes may (1) be more useable than unsimplified medical-language notes, (2) improve the patient-clinician relationship, and (3) empower patients through an enhanced understanding of their health care.

**Conclusions:**

AI software may translate medical notes into plain-language notes that are perceived as beneficial by patients. Limitations included sample size, inpatient-only setting, and possible confounding factors. Larger studies are needed to assess comprehension. Insight from patient responses to guided interviews can guide the future study and development of this technology.

## Introduction

Educating patients has been found to empower them to be involved participants in their health care and is an important principle of patient-centered care, yet clinicians are faced with increasing demands on their time, limiting opportunities to deliver thorough and understandable explanations to patients during encounters. Competing factors such as regulatory burden, documentation and billing, administrative responsibilities, and market forces that pressure clinicians to see more patients have increasingly limited time available to review medical information with patients. A study of ambulatory clinicians across four different specialties found that for each hour doctors spent at the bedside with patients, they spent 2 hours on desk work during the day, not including additional time spent documenting at home [1]. Another study of primary care physicians across 10 clinics found that more time is spent working in the electronic health record than on face-to-face interactions with patients [2].

Patients have rated communication and the quality of explanations about their health care among the most important factors for their satisfaction. In the inpatient setting, a recent focus group study of patients across four hospitals found that good communication and high-quality information provided at arrival and discharge were valued as important by patients [3].

Furthermore, improving patients’ knowledge about their conditions has been shown to improve patient compliance and clinical outcomes in various chronic disease models by affecting patient behavior [4]. The quality of explanations and patient understanding have been shown to directly affect health outcomes [5]. As one of many examples, a 2-year prospective study with 4341 patients found that hurried communication and fewer explanations were highly correlated with poor insulin adherence and diabetes-related complications [6].

OpenNotes emerged as a potential strategy to help educate patients about their health beyond the office visit. OpenNotes was launched as a research initiative in 2010 exploring the sharing of medical notes with patients that included 105 volunteer primary care physicians and their patients in Boston, rural Pennsylvania, and inner-city Seattle. Of 13,564 patients with visit notes available, 11,797 opened at least one note, and of those, 5391 completed a postintervention survey. This initial study found that 99% of patients who completed the surveys wanted the practice of sharing medical notes with patients to continue, with 85% stating that they would factor this into their decision in choosing future health care. Patients cited an improved understanding of their health and more trust in their clinicians [7]. A follow-up to this study was published in 2019 reporting the results of an online survey of 28,782 patient respondents. Among the 22,947 who reported reading one or more notes, 73% rated the notes as being very important in taking care of their health [8].

Many additional studies with OpenNotes have since been published and further reported on the benefits patients and clinicians have experienced when medical notes have been shared with patients. In one such study, 6913 patients completed a survey, and shared decision making was quantitatively measured. Researchers found a correlation between note reading and patient perception that they were participating in shared decision making [9]. In another study, adult patients and their care partners were surveyed before and after 12 months of exposure to their doctors’ notes electronically. At follow-up, patients stated they felt more confident in managing their health, felt better prepared for office visits, and had an improved understanding from baseline [10].

Despite the success of OpenNotes in research studies, a significant barrier to the widespread adoption of this approach has been clinician concerns that most patients may be confused by the extensive medical terms, abbreviations, and shorthand in medical-language notes [11]. Clinicians worry that the potential benefits of sharing medical notes may be offset by the need to spend more time clarifying these notes with patients or changing note-writing practices [12,13].

A strategy to bring OpenNotes to patients that does not significantly cost the clinician additional time is needed to encourage its adoption and may allow more patients to experience the benefits of having access to their medical notes.

In this study, we explored a method that may cost minimal time to clinicians through the application of recent advances in artificial intelligence (AI) technology. This approach harnesses machine learning and natural language processing (NLP) to simplify clinician written notes into plain-language notes. NLP is a branch of computer science that allows computer programs to interpret and manipulate human language and was shown to simplify medical texts as early as 2010 [14]. In contrast to methods such as the manual simplification of notes, which would require clinicians to spend time writing additional notes for patients, AI software can directly simplify medical language from existing systems. This may allow clinicians to preserve their note-writing practices, while creating a line-by-line plain-language version of patient notes. Such a note can be quickly reviewed by clinicians for correctness and may be more accessible for patients.

The application of NLP to simplify patient education materials has been an emerging field of research in recent years. Prototype models and applications of this technology have been created and conducted by various groups. A 2013 group in Romania created a model that increased the accessibility of medical language in a tele-care program by simplifying texts assigned by medical personnel into plain language [15]. A 2018 multisystem study assessed physician perception of the usability and quality of a web-based NLP system that linked medical notes in electronic health records to lay definitions. Physicians found that the system was easy to use with adequate lay definitions and recommended further development [16].

An important next step in establishing the utility of this approach has been to assess patient perceptions of plain-language notes. It has been important to identify whether patients see benefits of plain-language notes over medical-language (unsimplified) notes at all, and if so, how to best implement this practice. Therefore, our pilot study was designed to assess patient perceptions of AI simplified notes. We looked at whether patients find such notes to be useful and the benefits, concerns, or suggestions they may identify.

## Methods

### Materials Development

First, a synthetic patient note was generated with Synthea, an open-source, validated, software that generates synthetic patient records for research purposes [17]. The decision to use a synthetic note was made as synthetic notes offer the same utility of real patient notes without the associated privacy concerns. The note produced with Synthea simulated a real patient’s hospital discharge summary. Sections generated in this note included the admission diagnosis, other diagnoses, past medical history, hospital course, discharge disposition, plan, and discharge medications. This note served as the medical-language note for all parts of the study.

Proprietary AI software was then developed by a private company, AIPiphany, was used to simplify this medical-language note into a plain-language version [18]. The software replaced complex medical language with plain language equivalents and corrected for grammar and syntax. As an example, the statement “A chest computed tomography (CT) was done on 6/23/04 which showed no aortic dissection” in the original note was simplified by the program to “A chest CT was done on 6/23/04. This showed no tear in the inner layer of the large blood vessel of the heart.” This served as the plain-language note for all parts of the study. The parts of the note that were simplified included the history of present illness, past medical history, and hospital course. The decision to not simplify other parts of the note listed in the discharge summary was made as these portions were already generated with a patient as the intended reader, and further alteration of this text was deemed unnecessary and potentially confusing.

Notes were simplified by the AI software to a target Flesch-Kincaid measure of 5th grade level reading language. Although this was set as a target for the program, the actual Flesch-Kincaid measure of the note, not including the medication list, was 8th grade level reading language. The Flesch-Kincaid scale is a gold standard measure of readability that uses average sentence length (ASL) and average number of syllables per word (ASW) to approximate a grade level score using the following formula: (.39 × ASL) + (11.8 × ASW) – 15.59 [19]. Word version 16.0 (Microsoft Corp) was used to determine the Flesch-Kincaid measure of the notes. A 5th grade level reading language was selected as a target for the software as the average Medicare beneficiary reads at or below a 5th grade reading level [20]. This level may have made the simplified note accessible to as many participants as possible. However, while this was set as a target, this was not set as a hard constraint for simplifications. Under these conditions, the software attempted to simplify the note as close to a 5th grade level reading score as possible without eroding the meaning of the note. This produced a note of around an 8th grade level reading score as measured by the Flesch-Kincaid scale. This is likely both a limitation of the Flesch-Kincaid scale and the software used. While simplification of medical notes may decrease the average number of syllables per word, it requires using more words overall to convey the same meaning. This increases the average sentence length, reflected as an increase of the Flesch-Kincaid score. Additionally, simplifying notes further while still preserving the meaning and grammatical correctness of the medical note may require more robust changes to syntax and sentence structure than was possible with the software at the time of study. As the average US resident reads at or below an 8th grade reading level, this level of simplification was found to still be useful and relevant for our study purposes [20]. The simplified plain-language note was reviewed by an attending physician to check that the medical facts represented in the original note were not misrepresented in the simplified version. Other than editing font and formatting for print, no changes were made to the content of the simplified note.

It is important to note that participating patients were not tested with their own medical notes but rather those of the standardized synthetic patient generated with Synthea. This decision was made to prevent differences in complexities of patients’ health care from confounding the readability of the notes. By using the same pair of notes for all participants, standardization of the test material was achieved.

Two attending hospitalists reviewed the medical-language note generated with Synthea and shared a list of points they felt would be the most important for patients to understand. This was used to draft 7 reading comprehension questions through discussion and review by the hospitalists. Questions were written in multiple choice format with 4 options per question. Each question had one correct answer choice, two incorrect choices, and one choice stating, “I don’t know.” Patients were instructed to select “I don’t know” rather than guessing when unsure in order to reduce randomly correct answers. As an example, a question asks, “What is a concern if this patient suddenly stops taking coumadin?” Choices for this question were “The patient could have a blood clot that blocks a vessel,” “The patient could have a severe bleed,” “The patient would have problems with high blood pressure,” and “I don’t know.” The assessment questionnaire was also targeted to be at or below a 5th grade level by Flesch-Kincaid measure. The final questionnaire was found to have a Flesh-Kincaid score of 4.2, which meets our criteria. For reference, the comprehension assessment questions that were used are listed in Multimedia Appendix 1.

A demographic survey was also prepared, with selections for participants to record their gender, age, ethnicity, highest level of education, and health literacy as measured by a 2-item literacy screener (TILS). The TILS score is a self-reported score of health literacy from 1 to 5, with 5 reflecting the greatest level of comfort with reading medical documents and 1 being the lowest [21]. This method of assessing health literacy is quick to complete, minimizes participant embarrassment, and predicts health literacy on par with other commonly used but lengthier measurements [22]. These metrics were measured to assess for possible confounding effect on the comprehension score. All patient materials including the notes, comprehension questionnaire, and demographic survey were formatted similarly and printed on letter size paper in 12-point Times New Roman font.

Last, a set of questions and prompts was prepared for use by the researcher during a guided interview. Questions were designed to understand patients’ past exposure to OpenNotes, determine usability and usefulness of simplified notes, and identify suggestions patients may have to improve such notes. Three broad open-ended questions were written to be asked of all patients: “Have you ever read the notes that your doctor took about your visit?” “What did you think of these [simplified] notes?” “What else would you like to see in these [simplified] notes?” The complete list of guided interview questions with prompts can be found in Multimedia Appendix 2.

### Recruitment

Potential study participants were identified from patients hospitalized at the University of Colorado Hospital in July 2018. This study was approved by the Colorado institutional review board. Inclusion criteria were age 18 to 89 years, admitted to an internal medicine service, able to provide informed consent, and had been hospitalized for at least 48 hours. Exclusion criteria included incarcerated patients, pregnant patients, and patients who were not fluent in English to avoid language as a confounding factor in the study.

**Figure 1:**
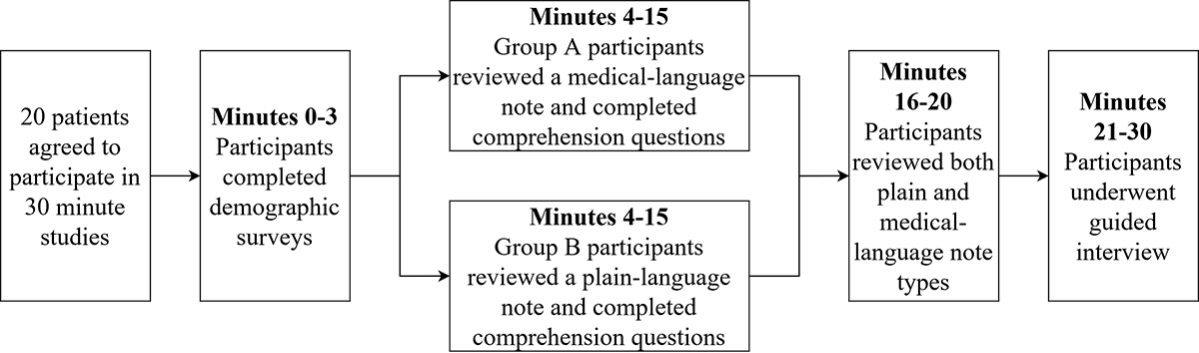
Participant assessment and interview flowchart.

Patients were consented, and studies were conducted in patients’ private hospital rooms. The purpose, procedures, risks, and benefits of the study were explained to participants, who were given sufficient time to review consent forms and ask any questions. To be respectful of participants’ time, the duration of the interview was limited to 30 minutes, enough time for the quantitative and qualitative sections of the study. A breakdown of times is seen in Figure 1. Three minutes were given at the start to allow participants to complete the demographic survey. Participants self-reported their gender, age group, ethnicity, highest level of education, and health literacy as measured by the TILS. These metrics were measured to assess if they had ancillary effect on the comprehension score and therefore could be confounding variables.

Participants were randomly assigned to one of two equal sized groups via computer generated sequence of note types. Group A participants read the medical-language note first and were tested on that note. Group B read the AI-generated plain-language version first and were tested on that simplified version. Participants were given 12 minutes to read their respective note and answer 7 reading comprehension questions.

All participants were then given both versions of the note and allowed 5 minutes to view the differences between the medical-language note and the simplified plain-language note. This was followed by a 10-minute guided interview to elicit their perception of the simplified note. A list of guided interview questions with corresponding prompts was used. When necessary, additional questions were asked to clarify patient statements. Participant responses to interview questions were transcribed electronically in text format as verbatim quotes or summarized statements. Statements by participants were only summarized if they were difficult to understand if transcribed exactly as spoken.

### Statistical Analysis

All quantitative analyses were performed using SPSS Statistics version 24 (IBM Corp). A Student *t* test was performed to assess if there was a significant difference in the mean comprehension assessment score between the group evaluated with the original medical-language note and the group evaluated with the simplified plain-language note. A multiple linear regression was performed to determine if the collected demographic variables may have been associated with the number of comprehension questions answered correctly.

### Thematic Analysis

Responses to guided interviews were coded as perceived benefits of simplified notes, perceived concerns about simplified notes, patient suggestions regarding simplified notes, and other insight. These responses were analyzed for recurrent themes.

## Results

### Characteristics of Participants

Of 34 patients approached about participation in the study, 20 agreed to participate. Those who agreed to participate completed all parts of the study, including the comprehension assessment and guided interviews. Reasons shared by participants for declining to participate included tiredness, disinterest, and a feeling of insufficient English language proficiency. Participant demographics are shown in Table 1.

### Statistical Analysis

The mean comprehension assessment score was found to be higher in the plain-language note group at 4.7 questions answered correctly compared with 3.9 questions answered correctly in the medical-language note group with a maximum possible score of 7. However, statistical analysis of this difference was limited by insufficient power of the Student *t* test (16%, α=.05) to detect if this difference was significant or not. Only one participant from each group answered all questions correctly. A multiple linear regression was performed to determine if the collected demographic variables of age group, gender, ethnicity, highest level of education, and health literacy may have been associated with the number of comprehension questions answered correctly. In multivariate modeling, health literacy measured as TILS score (*P*=.003), age (*P*=.03), and ethnicity (*P*=.03) were significantly associated with the comprehension assessment score (Multimedia Appendix 3).

**Table 1:** Study demographics (n=10).

Characteristics		Tested with unsimplified notes	Tested with simplified notes
**Gender, n (%)**			
	Male	7 (70)	2 (20)
	Female	3 (30)	8 (80)
**Age in years, n (%)**			
	18-34	7 (70)	7 (70)
	35-55	1 (10)	2 (20)
	55-64	2 (20)	0 (0)
	65+	0 (0)	0 (0)
**Ethnicity, n (%)**			
	White	7 (70)	7 (70)
	Black	1 (40)	2 (20)
	Hispanic	2 (20)	0 (0)
	Asian	0 (0)	0 (0)
	Other	0 (0)	1 (10)
**Highest education, n (%)**			
	Some high school	0 (0)	0 (0)
	High school diploma	2 (20)	1 (10)
	Some college (some credit with no degree or trade, vocational, or technical training)	5 (50)	6 (60)
	College graduate (associate degree, bachelor’s degree, or equivalent)	2 (20)	3 (30)
	Postgraduate (master’s degree, doctoral degree, or equivalent)	1 (10)	0 (0)
**Health literacy, mean (SD)**			
	TILS^a^ score	3.85 (1.05)	4.2 (0.82)

^a^Two-item literacy screener.

### Thematic Analysis

Three major themes of perceived potential benefits of simplified notes were found in guided interview responses: (1) enhanced usability of simplified notes compared with unsimplified notes, (2) improved patient-clinician relationship, and (3) empowerment of patients through an enhanced understanding of their conditions and management.

Regarding usability, participants shared that having access to a simplified note after a medical encounter would help them to better understand the information discussed during their visit.

...sometimes doctors use language that they don’t realize is above our level, so having something simple to take home would help.

Participants suggested that such notes may also help patients retain information discussed during the visit.

Things can get lost during a medical visit because you might be anxious or they [the clinician] may be talking too fast. Having a note like this to reference later is very useful.

Participants reported that while they often only skim through papers with medical terminology, they would take the time to read through a note that is written in plain language.

I only skim through or glance my own medical notes, but I would actually read through a simplified note.

The simplified version was much easier to understand. It was less confusing and helped me understand the procedure.

Regarding improved patient-clinician relationship, participants expressed that being given plain language notes would help them to have more trust and confidence in their clinician.

This [simplified] note would help because it would show me that the physician cared that I understood what was going on.

Patients stated that they would also be more likely to follow through with treatment recommendations due to this improved relationship.

...if I understood why my doctor wanted me to come to make an appointment, I would go.

Regarding patient empowerment, participants suggested that such notes would help them meet needs related to daily living by allowing them to more effectively communicate about their limitations with family members or employers. A participant shared that while he currently depends on family members to understand his medical management, having a plain-language note may reduce the need to ask others for assistance and therefore make him feel like less of a burden. Participants also shared that such notes would reduce the number of online searches they may need to conduct to understand their conditions. Another participant reported that such notes can also help empower patients to talk with their families about their problems.

It’s hard to talk to family when it’s in the doctor’s language but would be a lot easier with this.

Participants shared that employers may be more likely to understand the circumstances of patients and be responsive to their needs if patients are better able to explain their medical conditions.

This may help in getting paid time off work or breaks during shifts for medical reasons.

Another patient stated that these notes may allow him to overcome his hearing disability. He stated that he finds any form of text-based communication to be preferable to calling or talking in person due to his difficulty hearing spoken language. He stated that he would find it easier to clarify his questions by reading a text at his own pace rather than asking the clinician questions.

Other insight gained from interviews highlighted patients’ previous experiences with OpenNotes or reflected opportunities to improve plain-language notes. A patient shared that she has had previous exposure to OpenNotes as her primary care physician posts a plain-language summary alongside her postencounter documents on an online portal to which she has access, and she states that she found this useful to remember information from and better understand her medical visits.

Asked if they would prefer the original medical language, the simplified plain language, or both notes provided to them during visits, many participants reported that they wanted to have both copies. A participant shared that this may allow him to compare the two notes to see if he had missed anything by only reading the plain-language note. Another patient stated that she would also like to have both the medical-language and plain-language notes so she could use the simplified plain-language note for her own understanding but have the medical-language note to share with specialists or new clinicians.

Concerns reported by participants included possible oversimplification of medical language. Participants reported concerns that if notes are oversimplified, the physician’s intent may be lost. Patients also suggested that the level of simplification of notes would be best if adjusted to their level of experience with medical language. Participants additionally noted the opportunity to enhance the readability of simplified notes by reducing the length or repetition of some plain-language phrases.

## Discussion

### Principal Findings

The key findings of this pilot study are that (1) the main benefits noted by participants were an improved relationship with the clinician, increased usability of OpenNotes, and empowerment in their daily life; (2) patients desired access to their medical records and felt that simplified open medical notes would help them to better manage their health; and (3) while there was insufficient power to detect a significant difference in mean comprehension assessment scores, AI-simplified medical notes had been well received by patients.

### Limitations

As this was a pilot study with a small sample size (n=20), statistical analysis was limited by insufficient power of the Student *t* test (16%, α=.05) to detect a significant difference in comprehension scores between the medical-language and plain-language note groups.

Additionally, multivariate modeling had found that the TILS score, race, and ethnicity had a significant impact on the mean comprehension scores. These factors may have been possible confounders and should be controlled in future studies if assessing comprehension.

Given the small sample size, it is also possible that complete thematic saturation for guided interviews may not have been achieved. Additionally, although most Americans read at an 8th grade reading level, most patients enrolled in Medicare read at a 5th grade level or below. This may explain why only 2 out of 20 patients answered all questions correctly; it is possible that even the simplified notes were beyond participant reading levels.

Participants were also not provided their own medical notes but rather those of a standardized patient. This may have impaired participants’ ability to comprehend the notes due to a baseline lack of knowledge regarding the medical problems presented in the standardized note. As the plan and medications list from the patient discharge note were left unsimplified in this study, simplifying these portions of the note in future studies may be of further benefit for patients. Additionally, only one pair of notes was tested in this study. As future studies test additional notes, it is possible they may find note-specific variances that affect patient comprehension and perception.

Only an English-language note was tested, so our findings may not be generalizable to non-English applications. Last, all notes were simplified to the same reading level in this study. Individualizing the level of simplification of the notes to participant level of health literacy may enhanced reader comprehension. Another possible source of confounding that has not been directly measured is patients’ prior exposure to OpenNotes, as having had practice with reading notes may better equip them to read medical notes.

### Comparison With Previous Work

Institutions around the country have begun large-scale implementation of OpenNotes with encouraging responses from patients and clinicians. Preliminary studies have shown that computer technology may be used to simplify medical language to assist in patient understanding of medical terminology. However, this is the first known study to assess patients’ perception of medical notes that have been simplified with AI software. Patients’ responses in this study may be useful for the development of this work and offer a glimpse into the future of how patients may be able to improve their understanding outside of the medical visit.

### Future Studies

This is an interesting and emerging field of research with many opportunities for future study. As this initial pilot study found that patients perceived AI-generated simplified medical notes as desirable and useful, future studies should be conducted with larger sample sizes and take advantage of patients’ insights and suggestions as mentioned here. In future studies, patients’ own notes should be tested, and patients of different levels of care complexity should be included, as it is possible that patients with more complex management may derive a greater benefit from plain-language explanations. Studies should be repeated in the outpatient setting with longer exposure and follow-up times. The value of simplifying additional parts of the note such as the plan and medications list should be explored. Physicians should be surveyed on their perceptions of plain-language notes to assess if such notes would make them more likely to use OpenNotes in their own practices.

Suggestions from this study can be used to improve the NLP software used to simplify notes for future studies. As AI technology and use in this area evolves, methods should be developed to match the patient’s health literacy, cultural and demographic background, and level of health care experience with the simplification level of the note. Methods should be developed to reduce lengthy plain-language phrases. At the same time, care should be taken to ensure that the simplification process does not lead to omission of information that physicians perceive as valuable for patient understanding and utility. Long-term studies should evaluate the impact of plain-language OpenNotes on clinical outcomes in various settings.

### Conclusions

In conclusion, this study suggests that AI software may be used to generate plain-language medical notes that patients desire and find useful. Such notes may empower patients to better communicate and make decisions, increase adoption of OpenNotes, enhance the patient-clinician relationship, and improve clinical outcomes. The findings in this study can be used to optimize delivery and generation of simplified notes. Researchers in this field may particularly find the patient responses in the guided interviews in this study to be interesting and useful for the further development and application of NLP software in this field.

## Appendix

Multimedia Appendix 1 Comprehension assessment questionnaire.

Multimedia Appendix 2 Guided interview questions.

Multimedia Appendix 3 Statistical analyses.

## Abbreviations

AI: artificial intelligence
ASL: average sentence length
ASW: average number of syllables per word
CT: computed tomography
NLP: natural language processing
TILS: two-item literacy screener

## References

[1] Sinsky C, Colligan L, Li L, Prgomet M, Reynolds S, Goeders L, Westbrook J, Tutty M, Blike G (2016). Allocation of physician time in ambulatory practice: a time and motion study in 4 specialties. Ann Intern Med.

[2] Young RA, Burge SK, Kumar KA, Wilson JM, Ortiz DF (2018). A time-motion study of primary care physicians' work in the electronic health record era. Fam Med.

[3] Rapport F, Hibbert P, Baysari M, Long JC, Seah R, Zheng WY, Jones C, Preece K, Braithwaite J (2019). What do patients really want? An in-depth examination of patient experience in four Australian hospitals. BMC Health Serv Res.

[4] Gold DT, McClung B (2006). Approaches to patient education: emphasizing the long-term value of compliance and persistence. Am J Med.

[5] Marcus C (2014). Strategies for improving the quality of verbal patient and family education: a review of the literature and creation of the EDUCATE model. Health Psychol Behav Med.

[6] Linetzky B, Jiang D, Funnell MM, Curtis BH, Polonsky WH (2017). Exploring the role of the patient-physician relationship on insulin adherence and clinical outcomes in type 2 diabetes: insights from the MOSAIc study. J Diabetes.

[7] Delbanco T, Walker J, Bell SK, Darer JD, Elmore JG, Farag N, Feldman HJ, Mejilla R, Ngo L, Ralston JD, Ross SE, Trivedi N, Vodicka E, Leveille SG (2012). Inviting patients to read their doctors' notes: a quasi-experimental study and a look ahead. Ann Intern Med.

[8] Walker J, Leveille S, Bell S, Chimowitz H, Dong Z, Elmore JG, Fernandez L, Fossa A, Gerard M, Fitzgerald P, Harcourt K, Jackson S, Payne TH, Perez J, Shucard H, Stametz R, DesRoches C, Delbanco T (2019). OpenNotes after 7 years: patient experiences with ongoing access to their clinicians' outpatient visit notes. J Med Internet Res.

[9] Fossa AJ, Bell SK, DesRoches C (2018). OpenNotes and shared decision making: a growing practice in clinical transparency and how it can support patient-centered care. J Am Med Inform Assoc.

[10] Wolff JL, Darer JD, Berger A, Clarke D, Green JA, Stametz RA, Delbanco T, Walker J (2016). Inviting patients and care partners to read doctors' notes: OpenNotes and shared access to electronic medical records. J Am Med Inform Assoc.

[11] Bell SK, Mejilla R, Anselmo M, Darer JD, Elmore JG, Leveille S, Ngo L, Ralston JD, Delbanco T, Walker J (2016). When doctors share visit notes with patients: a study of patient and doctor perceptions of documentation errors, safety opportunities and the patient-doctor relationship. BMJ Qual Saf.

[12] Delbanco T, Walker J, Darer JD, Elmore JG, Feldman HJ, Leveille SG, Ralston JD, Ross SE, Vodicka E, Weber VD (2010). Open notes: doctors and patients signing on. Ann Intern Med.

[13] Alpert JM, Morris BB, Thomson MD, Matin K, Geyer CE, Brown RF (2019). OpenNotes in oncology: oncologists' perceptions and a baseline of the content and style of their clinician notes. Transl Behav Med.

[14] Kandula S, Curtis D, Zeng-Treitler Q (2010). A semantic and syntactic text simplification tool for health content. AMIA Annu Symp Proc.

[15] Topac V, Stoicu-Tivadar V (2013). Patient empowerment by increasing the understanding of medical language for lay users. Methods Inf Med.

[16] Chen J, Druhl E, Polepalli Ramesh B, Houston TK, Brandt CA, Zulman DM, Vimalananda VG, Malkani S, Yu H (2018). A natural language processing system that links medical terms in electronic health record notes to lay definitions: system development using physician reviews. J Med Internet Res.

[17] Walonoski J, Kramer M, Nichols J, Quina A, Moesel C, Hall D, Duffett C, Dube K, Gallagher T, McLachlan S (2017). Synthea: an approach, method, and software mechanism for generating synthetic patients and the synthetic electronic health care record. J Am Med Inform Assoc.

[18] AIpiphany.

[19] Dubay W (2004). The principles of readability.

[20] Stossel LM, Segar N, Gliatto P, Fallar R, Karani R (2012). Readability of patient education materials available at the point of care. J Gen Intern Med.

[21] Brice JH, Foster MB, Principe S, Moss C, Shofer FS, Falk RJ, Ferris ME, DeWalt DA (2014). Single-item or two-item literacy screener to predict the S-TOFHLA among adult hemodialysis patients. Patient Educ Couns.

[22] Morris NS, MacLean CD, Chew LD, Littenberg B (2006). The Single Item Literacy Screener: evaluation of a brief instrument to identify limited reading ability. BMC Fam Pract.

