# Supplementary material for: Patient Perception of Plain-Language Medical Notes Generated Using Artificial Intelligence Software: Pilot Mixed-Methods Study
Source: JMIR Form Res. 2020 Jun 5;4(6):e16670. doi: 10.2196/16670 (PMC7305564; doi:10.2196/16670)
Supplement: Multimedia Appendix 1 [file formative_v4i6e16670_app1.docx]

# Appendix A

## **Comprehension Assessment Questionnaire**

| **Please circle the letter of the answer you think best answers the question:**  **1. Why was an angioplasty performed on this patient?**  A. To fix a blocked blood vessel.  B. To restart the heart.  C. To repair torn blood vessel.  D. I don't know.    **2. The patient is taking a medication called novalog to treat a condition. What else should the patient do to help manage this condition?**  A. The patient should keep track of their cholesterol.  B. The patient should keep track of their blood pressure.  C. The patient should keep track of their blood sugar levels.  E. I don't know.    **3. What is a concern if this patient suddenly stops taking coumadin?**  A. The patient could have a blood clot that blocks a vessel.  B. The patient may have a severe bleed.  C. The patient would have problems with high blood pressure.  E. I don't know.  **4. What imaging procedure did they perform in the patient’s abdomen during her hospital visit and why?**  A. They used X-ray scans to see if there was internal bleeding.  B. They used special dye and computer scans to see if blood flow was blocked.  C. They did an MRI to see if there were problems in her blood vessels.  D. I don't know. | **5. The patient has had valve replacement surgeries in the past. What is the most important concern for the patient because of this?**  A. The patient may have a problem with the pace-maker of the heart.  B. The patient may have an increased chance of blood clots.  C. The patient may get an infection in their lungs.  D. I don't know.    **6. Why would it be especially worrying if this patient has sudden neck, back, and chest pain, given her medical problems?**  A. She could be having a blood clot blocking a vessel.  B. She could be having a seizure.  C. She could be bleeding internally.  D. I don't know.    **7. Why do the doctor and coumadin clinic want to monitor the ‘INR’?**  A. INR is part of a test to see if the patient has inflammation.  B. INR is part of a test to see if the patient has a blood pressure problem.  C. INR is part of a test to see if the patient could have a problem with bleeding or blood clotting.  D. I don't know. |
| --- | --- |
