# Supplementary material for: Patient Perception of Plain-Language Medical Notes Generated Using Artificial Intelligence Software: Pilot Mixed-Methods Study
Source: JMIR Form Res. 2020 Jun 5;4(6):e16670. doi: 10.2196/16670 (PMC7305564; doi:10.2196/16670)
Supplement: Multimedia Appendix 2 [file formative_v4i6e16670_app2.docx]

# Appendix B

## **Guided Interview Questions**

**Script:** “Have you ever read the notes your doctor took about your visit?”

- If yes: How did you get ahold of the notes? Was it easy?
- If not: why?

**Script:** “What do you think of these notes?”

- Prompt: How easy was it for you to understand?
- Prompt: What might make it easier for you to understand it?
- Prompt: Would this type of note be helpful to you?
- Prompt: What did you notice about the two notes? How were they similar? How were they different? Did you like one more than the other?
- Prompt: How might receiving a note like this affect you and your Doctor?
- Prompt: Do you have any worries about giving patients notes like these? Why?

**Script:** “What else would you like to see in these notes?”
