# Supplementary material for: Patient Perception of Plain-Language Medical Notes Generated Using Artificial Intelligence Software: Pilot Mixed-Methods Study
Source: JMIR Form Res. 2020 Jun 5;4(6):e16670. doi: 10.2196/16670 (PMC7305564; doi:10.2196/16670)
Supplement: Multimedia Appendix 3 [file formative_v4i6e16670_app3.docx]

# Appendix C

## **Statistical Analyses**

**Appendix Table 1 Multiple linear regression reflecting the association between demographic variables and comprehension assessment questionnaire scores**

|  | Unstandardized Coefficients | | Standardized Coefficients | t | Sig. |
| --- | --- | --- | --- | --- | --- |
|  | B | Std. Error | Beta |  |  |
| Note Type | -.088 | .522 | -.026 | -.168 | .869 |
| TILS Score | 1.512 | .406 | .811 | 3.721 | .003 |
| Gender | .684 | .605 | .199 | 1.129 | .279 |
| Age Group | .379 | .153 | .400 | 2.472 | .028 |
| Ethnicity | .479 | .197 | .347 | 2.436 | .030 |
| Education | .045 | .196 | .041 | .230 | .821 |
